# Disruption and dislocation in post-COVID futures for digital health

**DOI:** 10.1177/2053951720949567

**Published:** 2020-08-20

**Authors:** Richard Milne, Alessia Costa

**Affiliations:** Society and Ethics Research Group, Connecting Science, Hinxton, UK

**Keywords:** Expectations, futures, space, digital health, COVID, timing

## Abstract

In this piece we explore the COVID pandemic as an opportunity for the articulation and realization of digital health futures. Our discussion draws on an engagement with emergent discourse around COVID-19 and ongoing work on imaginaries of future care associated with digital tools for the detection of cognitive decline and the risk of dementia. We describe how the post-COVID futures of digital health are narrated in terms of the timing and speed with which they are being brought into being, as market actors attempt to establish the scale and durability of the COVID transformation. However, we also point to the particularly spatial changes to medical practice they envisage. In a time of distancing and isolation, the ability to operate effectively at a distance has become integral to the future of medical assessment, diagnosis and care. However, spatialized promises of digital health and the ability to act remotely are unevenly spread – some organizations and entities inevitably have greater reach.

## Disruption and dislocation in post-COVID futures for digital health

For over two decades, policy announcements have emphasized ‘technology’s place in a paradigm shift in the conceptualization and organization of British health care’ (May et al., 2001: 1890). More recently, ‘digital health’ technologies employing consumer devices such as smartphones, computers and wearables have been associated with a further wave of hype and speculation, and the emergence of narratives of the technological disruption of healthcare (Lupton, 2018). As ‘the first pandemic of the algorithmic age’ (Kind, 2020), data inheres in every aspect of the response to COVID, from contact tracing, to symptom tracking and diagnosis. Enthusiasm for, and expectations of, telemedicine, digital health and associated data practices have been renewed and revitalized. Directly COVID-19 oriented applications such as contact-tracing apps are accompanied by digital approaches to the delivery of telecare, the use of data-driven digital health tools for the assessment of health and the management and detection of disease and the wider and more rapid sharing of health records.

In this essay, we explore the articulation and realization of digital health futures in relation to the COVID pandemic. We engage with emergent discourse around COVID-19 and our ongoing work on the imaginaries of future care associated with digital tools for the detection of cognitive decline and the risk of dementia. We describe how the post-COVID futures of digital health are narrated in terms of the timing and speed with which they are being brought into being, as market actors attempt to establish an opening for enduring change post-COVID. Additionally, we point to the techno-geographies (Oudshoorn, 2012) of digital health these transformations envisage and enact, and their encounters with the everyday spaces and scales of healthcare. In doing so, we point to the need for ongoing critical examination of the role of emergent and emergency health data practices in both ‘disrupting’ and ‘dislocating’ the clinic.

### Timing and speed for a digital future

Moments of technoscientific opportunity and the futures associated with them neither arise entirely on their own, nor have their own internal logic. Their narratives are ‘fictional’ – stories, theories and discourses that project of a future state of the world in such a manner that orients and motivates actors in the present (Beckert, 2013). As Beckert puts it ‘it is the future that shapes the present – or, to be more specific: it is the images of the future that shape present decisions’ (2013: 221). Needless to say, such orienting ‘fictions’ are important in times of uncertainty, when the future seems particularly unknowable. In a similar vein, work in the sociology of technological expectations has described how expectations of technological futures become performative, drawing into the present elements of the futures they depict, supporting certain courses of action and precluding others (Brown and Michael, 2003). Geiger (2020) explores how such techno-revolutionary imaginaries are central to the narratives and business models of digital health. She captures the ‘eschatological’ nature of digital futures, their tendency to work backwards from ‘a metaphorical future perfect’ and the almost ‘messianic’ identification of a unitary ‘techno-vision’ as the sole alternative to the status quo. For example, a 2018 report from British think-tank Demos references the narrative of impending doom in the popular TV series *Game of Thrones*, ‘Winter is coming, HealthTech is here’ (Jones, 2018), and points to the salvific use of technology to alleviate (pre-COVID) pressures on the UK health service from winter influenza epidemics.

Demos’ identification of the pressing need for HealthTech, and the perceived ability of technological solutions to provide a productive, disruptive response to the pandemic provides the grounding for a sense of *timing* in the contemporary construction of post-COVID futures for digital health. As work in studies of science and innovation shows, the characterization and construction of moments of opportunity in the present is crucial to the projection of futures. Not least, it relies on the ability of actors to marshal the rhetorical, narrative and material resources to establish the sense that this is the ‘right time’, a kairotic ‘opening’ for innovation, (Brown, 2000; Miller, 1994).

In the case of COVID, analysts from management consultancy Oliver Wyman describe how:

For years, we’ve been talking about telehealth’s promise. But we have strained to find the one trigger that would unleash consumer demand for it, and provider acceptance of it, as an essential modality … Now is the time for employers, health plans, and government sponsors to uniformly endorse telehealth and stream-line the path from patient to clinician. (Shellenbarger et al., 2020)

Other analysts suggest, ‘[t]eleheath is on the cusp of mass adoption as the COVID-19 pandemic intensifies’ (Trzcinksi et al., 2020). In addition to consultancies, digital health companies themselves have been quick to both recognize the potential for an opening and contribute to its definition. As one digital health company CEO describes,

I always propose telehealth to our investors and usually get the response that ‘maybe it’s not the time, let’s see what happens’. *Now the times are ready* (Fischer, 2020, our emphasis).

The second feature of current narrative of disruption is that of speed and acceleration; the future is upon us, advancing at a rate that requires us to react, respond and adapt. Given this, it is unsurprising to see commentaries on the impact and potential of wider technological responses to COVID-19 that capture this. Thus Microsoft CEO Satya Nadella has described how the world had seen ‘two years’ worth of digital transformation in just two months’ (Nadella, 2020), 23andMe founder Anne Wojcicki identifies the pandemic as a ‘pivot point’ for digital health (2020), while another health technology company CEO puts it as ‘10 years of digital health evolution in 10 days’ (Ascione, 2020). This acceleration is further facilitated by the action of governments and regulators endorsing the need for action at speed in a state of emergency. Governmental and regulatory responses to, the regulation of health data flows have emphasized flexibility in the use and sharing of clinical and digital data (Oliver et al., 2020). Regulatory responses have aimed to assuage clinician and research concerns about restrictions on COVID-related data practices, allowing the use of WhatsApp on clinicians’ own devices for sharing patient details (in the UK, NHSX, 2020), or providing for regulatory discretion with regards to HIPAA enforcement for consultations by Skype or Facetime in the USA (HHS, 2020). In addition, new flows of clinical data have been established by the shift in emphasis from the ‘core’, typically the data collection and storage systems of healthcare providers, to ‘the edge’, including consumer mobile and connected devices. These changes create concerns about the ability to secure and control data flows, as well as about the quality and equivalence of data collected across multiple devices - and to row back such changes in the future.

Although framed in the disruptive moment of the pandemic, COVID-inspired futures for digital health are neither newly conceived nor short term. As Geiger describes, pre-pandemic digital health narratives depict ‘nothing short of a full-scale revolution, led by a collection of (health) technology disruptors’ (2020: 176). Further, unlike temporary pandemic responses like social distancing, digital health companies and proponents describe (and perform) an enduring transformation in the delivery of healthcare and the collection and use of diverse data: ‘The move will usher in longer-term change for healthcare and the ecosystems surrounding it’ (Shellenbarger et al., 2020). Another industry commentary describes how, ‘COVID-19 heralds a golden age of telehealth’ (Amy-Vogt, 2020). Here, telehealth forms one element of a wider ‘data-driven’ approach to health care. Similarly, the regulatory flexibility established during the emergency is seen to offer a template for a future in which the governance of health data collection, storage and sharing looks quite different from the pre-COVID era (Brookings Institute, 2020)

Such data-centric practices are not, however limited to COVID itself, a temporary solution to the challenges presented by the coronavirus, but as a model to be extended to other, non-communicable diseases, as captured in the quote from Richard Fischer, of the digital cognitive assessment company Altoida, above. The openings for digital health created by coronavirus become propitious and productive moments for novel, data-driven diagnostic practices of the future, as emphasized further in an interview with a digital cognitive testing company in which the interviewee commented that:

For me what’s really exciting about this is the opportunity to expand access to cognitive testing for people. Neuropsychology has traditionally been a very in-person type of thing, where you’re taking these old-school pen-and-pencil tests … The field hasn’t really gotten with the time quite yet. And what I think is quite interesting is with this all COVID-19 situation, people are seeing the need for remote cognitive assessment. (interview with AC, 16 April 2020)

Here, rather than muddling through in the context of a pandemic, clinical practice in an emergency is being seen as a template for the future, data-centred delivery of care. However, the post-COVID vision is not created *de novo*, but extends an already-imagined future of health data practice. The move towards this future that privileges not only those patients who are better resourced and technologically adroit, but also those healthcare systems who are aligned with, and have invested in, this technological future. This is made clear in a *New England Journal of Medicine (NEJM)* commentary on telehealth for COVID-19, which describes how ‘health systems that have already invested in telemedicine are well positioned to ensure that patients with COVID-19 receive the care they need’ (Hollander and Carr, 2020: 1681). The post-COVID durability of digital health may thus be less of a transformation than a consolidation of a techno-clinical landscape. Specifically, it is a vindication of those who previously invested in one particular techno-vision, now elevated to be the only possible alternative to the pre-COVID world.

#### Extending and expanding the spaces of digital health

The temporal characteristics of this eschatological techno-vision are accompanied by its spatial aspects – a ‘dislocation’ that occurs alongside disruption. In the remainder of this piece, we suggest that concentrating on these dislocative elements of digital health narratives can complement analyses of surveillance and privacy to draw attention to the new and emerging spatial configurations and practices associated with digital health data.

Space is a central element of the elaboration of techno-scientific narratives for healthcare, in futures which anticipate a centrifugal movement of medical records and health data from the ‘core’ to distributed ‘edge’ systems of data collection and sharing. In the case of coronavirus, these spatial visions come together with the established spatial practices of power and control of infectious disease control. International responses to the pandemic thus combine quarantine instructions to ‘stay at home’ or ‘shelter in place’, with digital health tools which aim to identify, capture and disrupt the movement of people and viruses.

In the digital health response to the pandemic, ‘telehealth is the “new front door”’ of the clinic (Orcutt, 2020). In such techno-geographies (Oudshoorn, 2012) space is conceived as distance which can be condensed or done away with (cf Leszczynski and Crampton, 2016), reducing costs, engaging hard to reach communities and diminishing the burden on patients and families, who will be able to receive timely care from the comfort of their own home. The putative contribution of Google and Apple to contact tracing technology thus relies on an image of their place at the heart of a globalized, ‘placeless’ network, able to intervene in a topology of inter-personal interactions regardless of location. In clinical commentaries, the moving threshold of the clinic itself is the object of concern. In their *NEJM* piece Hollander and Carr, for example, describe healthcare practices reliant on algorithmic ‘forward triage’ in which ‘automated logic flows’ draw on clinical data along with information about travel, exposure and local epidemiology, enabling some patients and doctors to avoid physical clinic visits entirely, while prioritizing potential COVID cases (Hollander and Carr, 2020). These visions of tele-health in turn rely on extant and novel practices involved in the production, circulation and use of health-relevant data, and enable the ‘remote’ clinician to:

[V]isualize a patient’s medical history, every provider they have ever seen, where a patient resides, where they work, their demographic information, and other pertinent data and then generate a journey of activities or events of where patients are coming from. (Orcutt, 2020)

The clinical ‘core’ here, is able to draw in information from across its web of datasets. However, while the spatial constraints of medicine are ostensibly erased, the data practices described recapitulate not only a metaphorical patient ‘journey’, but a data journey grounded in the locales of healthcare and the practices of patients, clinicians and others.

A feature of the response to COVID has been the elaboration of digital tools that are co-produced with configurations of the scales of data collection, and with concerns about the ethics of COVID data practices. For example, contact tracing apps, such as those now implemented in France, Germany or South Korea, rely on adoption through a sense of solidarity, while their implementation, notably in China and Israel draws attention to the powers and capabilities of the state, while reinforcing these and raising significant and enduring concerns about surveillance and privacy (Kitchin, 2020). Elsewhere, Madrid’s CoronaMadrid app (CoronaMadrid, 2020), or the Parisian hospital system’s Covidom site (Covidom, 2020), operate at an urban scale, facilitating symptom reporting and remote patient monitoring, while establishing data citizenship as a response to *civic* health service concerns. In a final example, the implementation of Sensyne’s CVm-Health coronavirus tool (CVm-Health, 2020) detaches data practices from the national or urban environment, and situates them amidst the intimate and quotidian geographies of neighbourliness, in which health data collection enables and enacts neighbourly concern and care for the digitally disconnected.

The new arrangements of actors, places and tools associated with the digital health response to COVID thus present challenges in relation to surveillance, but also prompt us to consider how the practical and ethical configurations of data practices are co-produced with the scales and spaces of public health and infectious disease medicine. While the emerging geographies of post-COVID digital health are imagined as exempt from the limits of distance, this brief overview points to their emergence in dialogue with the scales, practices and situated ethical considerations associated with health data. In turn, these encounters between the present and future geographies of medicine have implications for what and where we consider clinical work, and indeed the ‘clinic’ to be, and, beyond that, the consequences of ways of medical working and seeing associated with novel data practices (cf Adams, 2017).

## Conclusion

In this piece, we have begun to consider the place of COVID-19 in imagining and constructing futures for digital health. We suggest that attending to the futures constructed for and by COVID data practices involves critically examining what is produced through narratives of speed and timing, and in relation to spatialized imaginaries of the future of care. In the process, we have drawn attention to the extent to which COVID is seen as an opportunity for durable change, which invokes novel data practices across a range of scales of social action. This, we suggest, involves not only dis-rupting, but also dis-locating the clinic. Examining and understanding these processes of disruption and dislocation is essential to interrogating the nature of health and wellbeing in a post-COVID world, and the acts of focusing and exclusion which may occur as the pressing ‘best we can do, here and now’ becomes the best we can aim for, anywhere, in the future.
